# Diverse organic-mineral associations in Jezero crater, Mars

**DOI:** 10.1038/s41586-023-06143-z

**Published:** 2023-07-12

**Authors:** Sunanda Sharma, Ryan D. Roppel, Ashley E. Murphy, Luther W. Beegle, Rohit Bhartia, Andrew Steele, Joseph Razzell Hollis, Sandra Siljeström, Francis M. McCubbin, Sanford A. Asher, William J. Abbey, Abigail C. Allwood, Eve L. Berger, Benjamin L. Bleefeld, Aaron S. Burton, Sergei V. Bykov, Emily L. Cardarelli, Pamela G. Conrad, Andrea Corpolongo, Andrew D. Czaja, Lauren P. DeFlores, Kenneth Edgett, Kenneth A. Farley, Teresa Fornaro, Allison C. Fox, Marc D. Fries, David Harker, Keyron Hickman-Lewis, Joshua Huggett, Samara Imbeah, Ryan S. Jakubek, Linda C. Kah, Carina Lee, Yang Liu, Angela Magee, Michelle Minitti, Kelsey R. Moore, Alyssa Pascuzzo, Carolina Rodriguez Sanchez-Vahamonde, Eva L. Scheller, Svetlana Shkolyar, Kathryn M. Stack, Kim Steadman, Michael Tuite, Kyle Uckert, Alyssa Werynski, Roger C. Wiens, Amy J. Williams, Katherine Winchell, Megan R. Kennedy, Anastasia Yanchilina

**Affiliations:** 1 Jet Propulsion Laboratory, California Institute of Technology, Pasadena, CA USA; 2 Department of Chemistry, University of Pittsburgh, Pittsburgh, PA USA; 3 Planetary Science Institute, Tucson, AZ USA; 4 Melanie Sauer and Associates, LLC, Sierra Madre, CA USA; 5 Photon Systems Incorporated, Covina, CA USA; 6 Earth and Planets Laboratory, Carnegie Institution for Science, Washington, DC USA; 7 The Natural History Museum, London, UK; 8 Department of Methodology, Textiles and Medical Technology, RISE Research Institutes of Sweden, Stockholm, Sweden; 9 Astromaterials Research and Exploration Science Division, NASA Johnson Space Center, Houston, TX USA; 10 Texas State University, Houston, TX USA; 11 Jacobs JETS II, Houston, TX USA; 12 Malin Space Science Systems, Inc., San Diego, CA USA; 13 Department of Geosciences, University of Cincinnati, Cincinnati, OH USA; 14 Division of Geological and Planetary Sciences, California Institute of Technology, Pasadena, CA USA; 15 Astrophysical Observatory of Arcetri, INAF, Florence, Italy; 16 Department of Earth and Planetary Sciences, University of Tennessee, Knoxville, TN USA; 17 Framework, Silver Spring, MD USA; 18 Department of Earth, Atmospheric, and Planetary Sciences, Massachusetts Institute of Technology, Cambridge, MA USA; 19 Department of Astronomy, University of Maryland, College Park, MD USA; 20 Planetary Geology, Geophysics and Geochemistry Lab, NASA Goddard Space Flight Center, Greenbelt, MD USA; 21 Blue Marble Space Institute of Science, Seattle, WA USA; 22 Department of Earth, Atmospheric, and Planetary Sciences, Purdue University, Lafayette, IN USA; 23 Department of Geological Sciences, University of Florida, Gainesville, FL USA; 24 School of Earth and Space Exploration, Arizona State University, Tempe, AZ USA; 25 Impossible Sensing, LLC, St. Louis, MO USA

**Keywords:** Astrobiology, Fluorescence spectroscopy, Raman spectroscopy, Geochemistry

## Abstract

The presence and distribution of preserved organic matter on the surface of Mars can provide key information about the Martian carbon cycle and the potential of the planet to host life throughout its history. Several types of organic molecules have been previously detected in Martian meteorites^1^ and at Gale crater, Mars^2–4^. Evaluating the diversity and detectability of organic matter elsewhere on Mars is important for understanding the extent and diversity of Martian surface processes and the potential availability of carbon sources^1,5,6^. Here we report the detection of Raman and fluorescence spectra consistent with several species of aromatic organic molecules in the Máaz and Séítah formations within the Crater Floor sequences of Jezero crater, Mars. We report specific fluorescence-mineral associations consistent with many classes of organic molecules occurring in different spatial patterns within these compositionally distinct formations, potentially indicating different fates of carbon across environments. Our findings suggest there may be a diversity of aromatic molecules prevalent on the Martian surface, and these materials persist despite exposure to surface conditions. These potential organic molecules are largely found within minerals linked to aqueous processes, indicating that these processes may have had a key role in organic synthesis, transport or preservation.

## Abstract (web summary)

Raman and fluorescence spectra, consistent with several species of aromatic organic molecules, are reported in the Crater Floor sequences of Jezero crater, Mars, suggesting multiple mechanisms of organic synthesis, transport, or preservation.

## Main

There are multiple origin hypotheses for the presence of organic matter on Mars from meteorite and mission studies. These include in situ formation through water–rock interactions^5^ or electrochemical reduction of CO_2_ (ref. ^6^), or deposition from exogenous sources such as interplanetary dust and meteoritic infall^1^, although a biotic origin has not been excluded. Understanding the fine-scale spatial association between minerals, textures and organic compounds has been crucial in explaining the potential pools of organic carbon on Mars. The Scanning Habitable Environments with Raman and Luminescence for Organics and Chemicals (SHERLOC) instrument is a tool that enables this on the Martian surface.

**Fig. 1:**
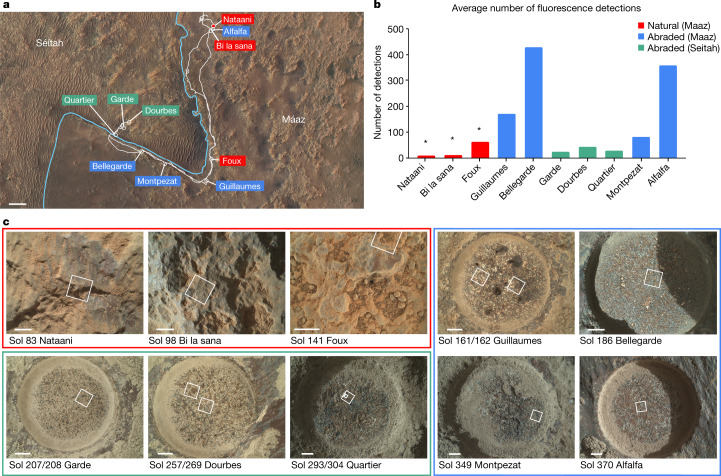
Overview of targets analysed by SHERLOC during the crater floor campaign. **a**, High Resolution Imaging ScienceExperiment (HiRISE) image of the region studied with the rover’s traverse marked in white, the boundary between the Séítah and Máaz fm delineated by the light blue line, and each rock target labelled. Scale bar, 100 m. **b**, Average number of fluorescence detections (out of 1,296 points) from survey scans for each target interrogated by SHERLOC, arranged in order of observation. *The acquisition conditions were different for dust-covered natural targets as compared to relatively dust-free abraded targets, possibly resulting in reduced detections. **c**, WATSON images of natural (red box) and abraded targets (Máaz is the blue box, Séítah is the green box) analysed in this study, with SHERLOC survey scan footprints outlined in white. Two survey scans were performed on Guillaumes, Dourbes and Quartier. Sol 141 imaging on Foux had an incomplete overlap of WATSON imaging and SHERLOC spectroscopy mapping. Scale bars, 5 mm.

**Extended Data Fig. 1:**
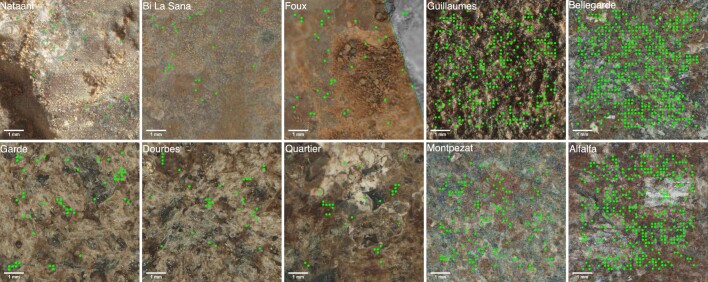
Group 2 Fluorescence Across All Targets Analyzed by SHERLOC. Colorized ACI images from survey scans of each of the 3 natural targets (top left), 4 Máaz abraded targets (right), and 3 Séítah abraded targets (bottom left). Green spots represent the relative laser beam diameter that have a positive identification for the ~335–350 nm fluorescence.

**Extended Data Fig. 2:**
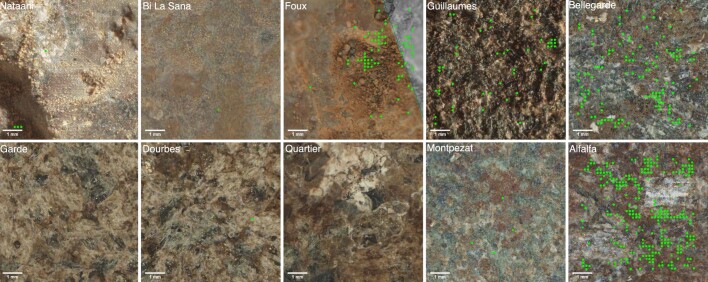
Group 3 Fluorescence Across All Targets Analyzed by SHERLOC. Colorized ACI images from survey scans of each of the 3 natural targets (top left), 4 Máaz abraded targets (right), and 3 Séítah abraded targets (bottom left). Green spots represent the relative laser beam diameter that have a positive identification for the ~270–295 nm fluorescence. No ~270–295 nm fluorescence was observed on survey scans in Garde and Quartier.

The Perseverance rover was designed for in situ science with the ability to collect a suite of samples for eventual return to Earth^7^. The rover’s landing site within Jezero crater combines a high potential for past habitability as the site of an ancient lake basin^8^ with diverse minerals, including carbonates, clays and sulfates^9^ that may preserve organic materials and potential biosignatures^10^. The Jezero crater floor includes three formations (fm)^11^; two of these, Máaz and Séítah, were explored as part of the mission’s first campaign. Máaz, previously mapped as the crater floor fractured rough unit, is highly cratered and broadly mafic in composition; rover observations to date indicate a composition rich in pyroxene and plagioclase^12^. Séítah, previously mapped as the crater floor fractured 1 unit, is underlying and therefore presumed older than Máaz and contains rocks that represent an ultramafic olivine-bearing cumulate^13^. SHERLOC has observed three natural (as found) rock surfaces in Máaz and seven freshly abraded surfaces across Máaz and Séítah (Fig. 1 and Extended Data Figs. 1 and 2). Abrasion consists of removing the outer layer of the rock, which is weathered and covered by Martian dust, using an abrading bit on the drill to create a 45 mm diameter cylindrical hole of 8–10 mm deep. The gaseous dust removal tool then removes residual fines with nitrogen gas^14^ to reveal a flat, dust-free surface for analysis. Four abrasion targets are associated with rock cores that may be returned to Earth during the Mars Sample Return campaign.

The SHERLOC instrument is a deep ultraviolet (DUV) Raman and fluorescence spectrometer designed to map the distribution of organic molecules and minerals on rock surfaces at a resolution of 100 μm (ref. ^15^). This approach enables spectral separation of weak Raman scattering from stronger fluorescence emission, which can have cross-sections that are 10^5^–10^8^ times larger than Raman^15^, allowing for measurement of both signals simultaneously. SHERLOC can detect Raman scattering from roughly 700 to 4,000 cm^−1^ and fluorescence photons from 253 to 355 nm (see Methods for more detailed descriptions). SHERLOC includes a autofocus context imager (ACI) coboresighted with the spectrometer to collect high spatial resolution (roughly 10.1 μm per pixel) grayscale images to place spectral maps within the context of texture and grain sizes. The wide-angle topographic sensor for operations and engineering (WATSON) imager provides colour imaging and broader spatial context. Combined, these enable spatial associations between organics and minerals to assess formation, deposition and preservation mechanisms. SHERLOC has previously observed fluorescence signatures consistent with small aromatic compounds in three targets across the crater floor^16^ that align with previous findings on Mars and within Martian meteorites.

## Fluorescence signals in the crater floor

**Fig. 2:**
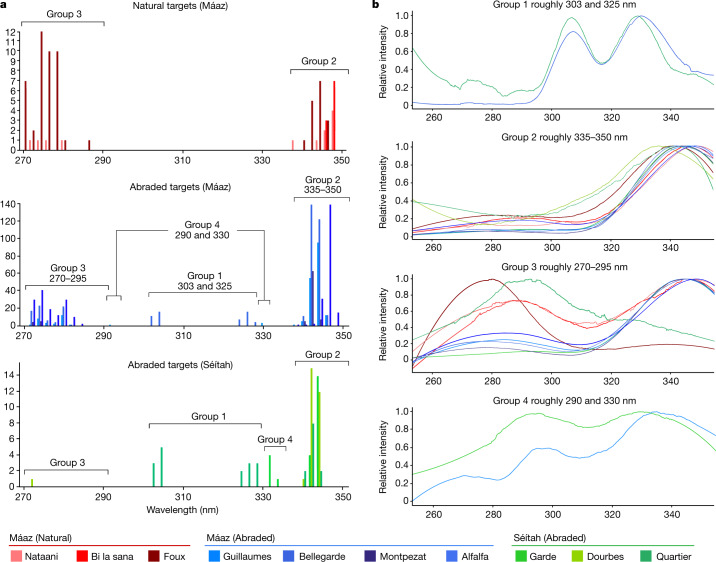
Summary of fluorescence features across targets. **a**, Histograms of the *λ*_max_ (measured from raw data) of four fluorescence features that were observed in survey scans in natural targets in Máaz (top, n = 84), abraded targets in Séítah (bottom, n = 82) and abraded targets in Máaz (middle, n = 1070). Bins of 1 nm show variation in band centres, *y* axes scaled to each dataset. **b**, Filtered mean spectra from each target representing each fluorescence feature category demonstrate characteristic band positions, normalized relative intensities and colocated features between targets. The range of the SHERLOC CCD is 250–354 nm. The rise in baseline below 270 nm is a boundary artefact introduced by the filter and not representative of the sample data^45^.

Fluorescence signals were detected on all ten targets observed by SHERLOC in the Jezero crater floor. They can be summarized by four main feature groups (Fig. 2). Group 1 is a doublet at roughly 303 and 325 nm; group 2 is a single broad band at roughly 335–350 nm; group 3 is a single broad band between roughly 270 and 295 nm and group 4 is a pair of bands centred at roughly 290 and 330 nm. The scan parameters are given in Extended Data Table 1. A two-sample Kolmogorov–Smirnov test was done on the observed fluorescence maxima for each group to determine whether they were statistically distinct from one another and found that groups 1–3 had null probabilities (likelihood that two groups are samples of the same distribution) of less than 10^−40^. Group 4 was too small for a statistical assessment, but is considered qualitatively different from the others.

**Extended Data Fig. 3:**
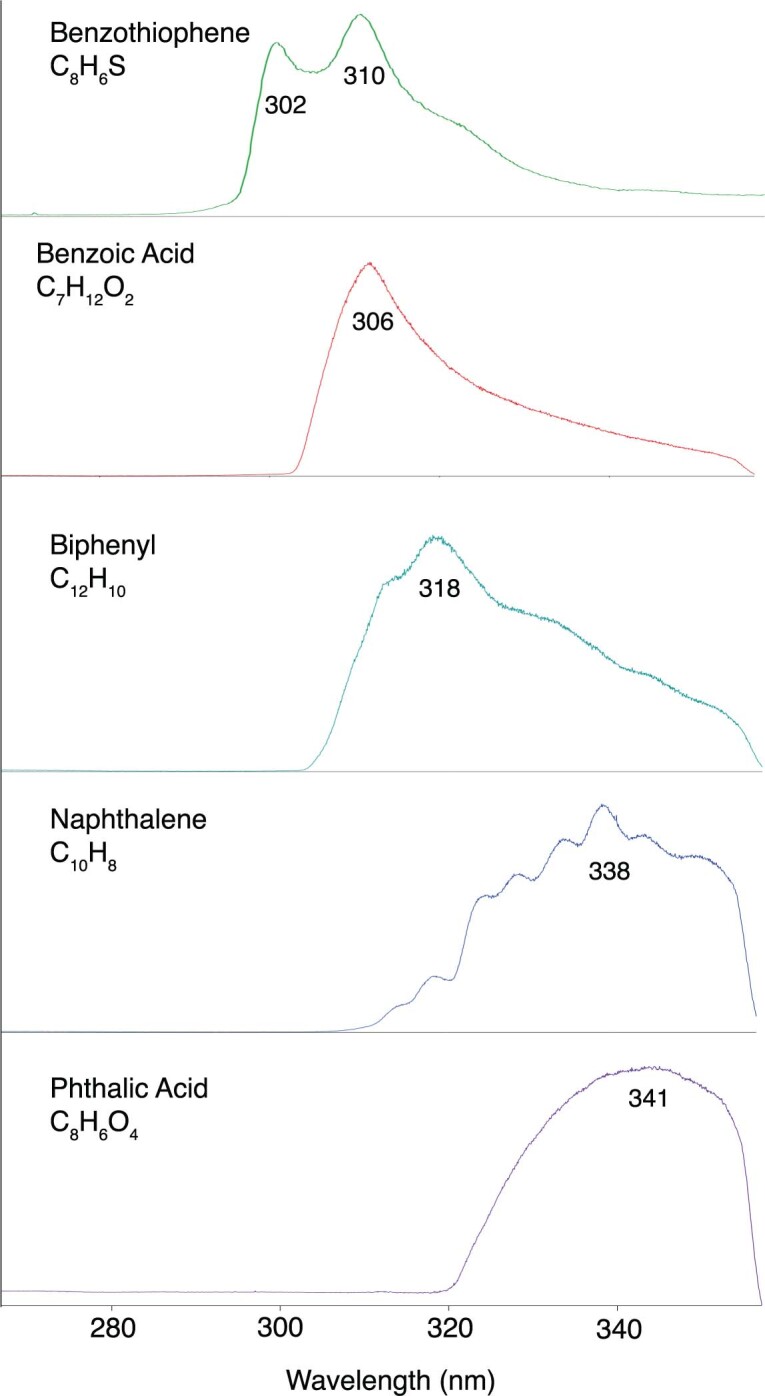
Reference Fluorescence Spectra for 1 and 2 ring organic compounds. Fluorescence emissions of a sample set of 1- and 2-ring organic compounds analyzed on a SHERLOC analog instrument.

The four fluorescence feature categories observed in the ten targets presented here are all consistent with emission in the spectral range shown by single ring aromatics and polycyclic aromatic hydrocarbons^15,17^ (Extended Data Table 2 and Extended Data Fig. 3). The number of rings in aromatic compounds can be estimated following the reported trend of emission spectra under DUV excitation^18^, in which increasing emission wavelength is positively correlated with number of aromatic rings; this was used to define the four fluorescence feature categories used in this study (Extended Data Table 3). However, the potential for non-organic luminescence^19^ must also be considered for each group and is discussed herein.

### Group 1: doublet at roughly 303 and 325 nm

**Fig. 3:**
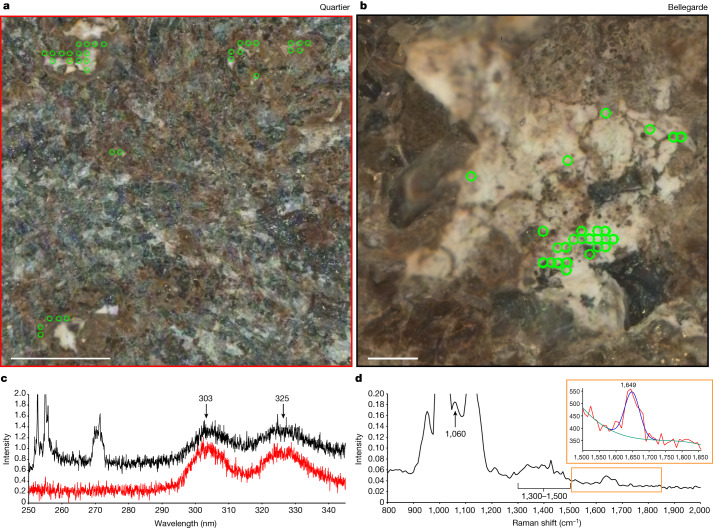
Group 1 (roughly 303 and 325 nm) doublet fluorescence feature mineral associations in Bellegarde and Quartier. **a**, Colourized ACI image of a region where a survey scan (36 × 36 points over 5 × 5 mm^2^) was performed on the Bellegarde target from sol 186. Green rings (rough laser beam diameter) represent locations where the roughly 303 and 325 nm fluorescence doublet was detected. **b**, Colourized ACI image of a region where a detailed scan (10 × 10 points over 1 × 1 mm^2^) was performed on the Quartier target from sol 304. Green rings represent locations where the roughly 303 and 325 nm fluorescence doublet was detected. Scale bars, 1 mm. **c**, Median fluorescence spectra (unfiltered) from the green points indicated in Bellegarde (red, n = 33) and Quartier (black, n = 26) normalized to 303 nm band and offset for clarity. **d**, Median Raman spectra of four points with highest fluorescence band intensities from Quartier scans on sols 293 and 304. Roughly 1,010 cm^−1^ sulfate band is off scale; inset shows roughly 1,649 cm^−1^ band with Voigt fit (FWHM 53.737, area 12,559, height 192.79). In the inset, the unfitted spectrum (red), fitted spectrum (blue) and baseline (green) are shown; *y* axis is intensity.

Two targets, Bellegarde and Quartier, showed the distinctive group 1 fluorescence feature (Fig. 3). These peaks appear together with constant relative positions and intensities, probably indicating a single emitter. The Bellegarde target, located on the Rochette rock in the Máaz fm, yielded detections on white crystals that are probably hydrated Ca-sulfate based on SHERLOC and PIXL observations^16^; the fluorescence doublet feature was associated with these areas (Fig. 3a,c). The Quartier target, located on the Issole rock in the Séítah fm, similarly contained white crystals that showed Raman peaks at 1,010–1,020 cm^−1^ and a broad band at roughly 3,500 cm^−1^ whose intensities were positively correlated (Fig. 3b,d). Sometimes, minor peaks at roughly 1,140 and at 1,215–1,225 cm^−1^ were also present. These peaks are consistent with a mix of sulfates^20^, potentially including both Ca- and Mg-sulfate at different hydration states. PIXL established that two different sulfate minerals were present, namely Mg-rich sulfate (66 wt% SO_3_, 27 wt% MgO, 3 wt% CaO, 4 wt% FeO) and CaMg sulfate (61 wt% SO_3_, 18 wt% MgO, 19 wt% CaO, 2 wt% FeO). A Raman peak at roughly 1,649 cm^−1^ was detected at one point within the hydrated sulfate crystal where doublet fluorescence was also present. This peak was accompanied by a small peak at roughly 1,050 cm^−1^ and a broader feature that seemed to contain several peaks between 1,330 and 1,410 cm^−1^ (Fig. 3d). Eleven sols later, several high-resolution scans subsequently performed on the same area of Quartier showed a nearly identical roughly 1,649 cm^−1^ peak at three points within hydrated sulfate crystals. In each case, the distinctive doublet fluorescence was detected as well as a broader feature at 1,330–1,410 cm^−1^.

The group 1 fluorescence observations in Quartier (Séítah) are consistent with the presence of a one or two-ring aromatic organic molecule(s) within a hydrated sulfate crystal. It is also possible that the observed emission comes from Ce^3+^ concentrated within the sulfate, given the close match in emission wavelengths in laboratory data. Three Raman peaks at 1,060, 1,330–1,410 and roughly 1,649 cm^−1^ are colocated with the three most intense doublet fluorescence and strong hydrated sulfate signals. They were detected even after 11 sols of surface exposure, although the hydration feature (OH stretch at roughly 3,300–3,500 cm^−1^) decreased in intensity, indicating a change in the hydration state after exposure to the Martian atmosphere. On the basis of the relative positions and intensities of these peaks, they represent at least two possibilities: vibrational modes of an organic molecule that include a preresonant C=C stretch^21^, or asymmetric stretching and bending modes from nitrate within the sample^22^. The possibility of organics occurring within sulfates is supported by evidence from studies of Martian meteorites^5^ and in Gale crater^21^, which show that sulfates may have a key role in forming, preserving or transporting organic molecules in the Martian environment. The combination of Raman and fluorescence data reported here could constitute two lines of evidence that support the detection of organic molecules within hydrated sulfate crystals, which is the simplest explanation for these observations. If both Raman and fluorescence signals are inorganic in origin, nitrate and Ce^3+^ in sulfate would need to be colocated.

### Group 2: single band at roughly 335–350 nm

**Extended Data Fig. 4:**
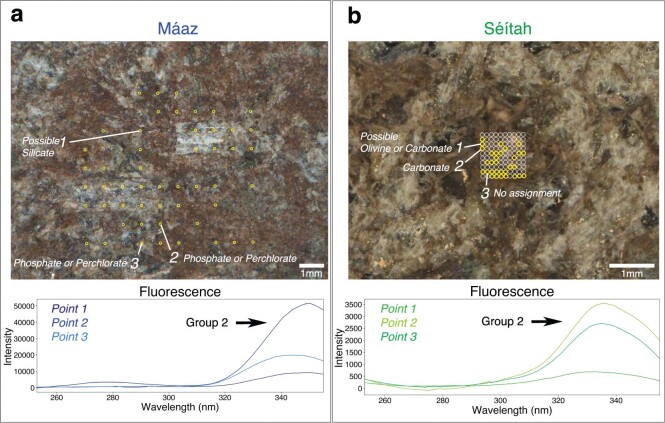
Group 2 Fluorescence Feature Mineral Associations in Alfalfa and Dourbes. A) Colorized ACI of Alfalfa (Máaz fm.) HDR scan on sol 370 with laser overlay (grey = no fluorescence detected, yellow = fluorescence at ~335–350 nm detected). Three points of interest with clear fluorescence are marked in white and correlate to the smoothed mean spectra below. Point 1 was co-located with Raman detections of amorphous silicate (broad ~1063 cm^−1^); Point 2 and 3 were co-located with potential perchlorate detections (~961 cm^−1^). B) Colorized ACI of Dourbes (Séítah fm.) Detail 1 scan on sol 269 with laser overlay (gray = no fluorescence detected, yellow = fluorescence at ~335–350 nm detected). Three points of interest with clear fluorescence are marked in white and correlate to the spectra below. Point 1 was co-located with Raman detections of potential olivine (~823 cm^−1^) and carbonate (~1077 cm^−1^); Point 2 was co-located with a potential carbonate detection (~10 cm^−1^); and Point 3 with an unassigned peak at ~1015 cm^−1^.

The most common fluorescence feature detected was a single broad (roughly 30–40 nm full-width at half-maximum (FWHM)) band centred at roughly 335–350 nm. Group 2 fluorescence was observed on all targets across both formations and showed the highest intensities among the four fluorescence feature categories (Fig. 2). The relative occurrence of this feature observed in survey scans of abraded targets was markedly higher in Máaz (189 ± 96 counts) versus Séítah (26 ± 6 counts). However, the average intensity of this feature was comparable between survey scans performed in the two formations (Máaz 342 ± 76 counts; Séítah 361 ± 80 counts). The measured intensity can vary on the basis of several factors, including the concentration of the emitter, the focus of the spectrometer and the presence of an absorbing material; therefore, large standard deviations are expected. Scans from all abraded targets show group 2 fluorescence detections that seem to be at or near grain boundaries in most cases (Extended Data Table 2 and Extended Data Fig. 1). In Máaz, the group 2 feature had an average band centre position of 344.1 ± 1.5 nm in survey scans and was observed to have band centres varying from roughly 338 to 349 nm, whereas in Séítah, the average band centre position was 343.1 ± 0.5 nm and the variance of the band centre had a narrower range, from roughly 340 to 345 nm (Fig. 2a). In abraded targets in both formations, the group 2 feature was associated with a common set of minerals detected with Raman spectroscopy, including carbonate, phosphate, sulfate, silicate and occasionally, potential perchlorate (Fig. 5 and Extended Data Fig. 4)^16,20^. The key difference in mineral associations was that in three Máaz fm targets (Montpezat, Bellegarde and Alfalfa), this feature was also associated with possible detections of pyroxene. By contrast, in the Séítah fm, this feature was associated with a possible detection of olivine in at least one point on each target (Extended Data Table 2).

**Fig. 4:**
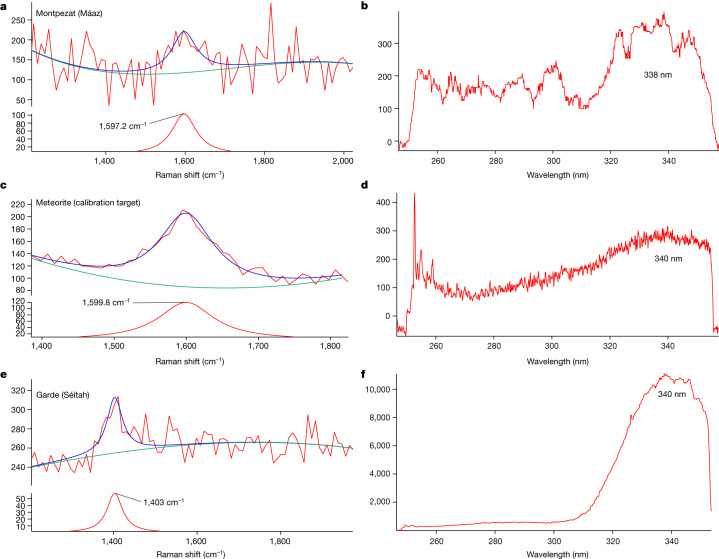
Raman features of possible organic compounds. **a**, Raman spectrum from point 40 of an HDR scan on Montpezat (sol 349) with a Lorentzian fit (FWHM 49.873, area 8,069.1, height 103). **b**, Corresponding average fluorescence spectrum to **a** (lambda max roughly 338 nm). **c**, Median Raman spectrum (n = 100) from an HDR scan on the SaU008 meteorite calibration target (sol 181), which contains the known graphitic (G) band, with a Lorentzian fit (FWHM 61.784, area 11,646, height 120). **d**, Corresponding average fluorescence spectrum to **c** (lambda max roughly 338 nm). **e**, Average Raman spectrum of points with the highest group 2 fluorescence (n = 28) on Garde (sol 207–208) with a Lorentzian fit (FWHM 47, area 4,500, height 60.953). **f**, Corresponding average fluorescence spectrum to **c** (lambda max roughly 340 nm). In all graphs, the unfitted spectrum (red), fitted spectrum (blue) and baseline (green) are shown; the *y* axis is intensity.

**Extended Data Fig. 5:**
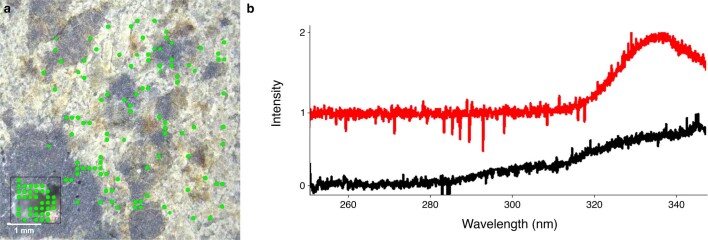
Mean Fluorescence Spectra on Two Regions of Meteorite Calibration Target. A) Colorized ACI image from a survey scan of the meteorite calibration target from sol 181. Green spots represent the relative laser beam diameter that have a positive identification for the ~335–350 nm fluorescence. B) Mean fluorescence spectra from locations by all green spots in A (red spectrum, n = 137) and mean fluorescence spectra from green spots bounded by the black box from the vug (black spectrum, n = 36).

One point in the high dynamic range (HDR) scan on Montpezat showed a Raman peak at 1,597 cm^−1^ as well as weak fluorescence at roughly 340 nm (Fig. 4a,b), and was colocated with a detection at roughly 1,080 cm^−1^. The roughly 1,080 cm^−1^ signal shows a broad-shaped Raman band consistent with laboratory studies of carbonate and silicate minerals^19^. Raman spectroscopy cannot resolve silicate phases well because of the small degree of polarizability of the silicon-oxygen tetrahedron^23^; therefore, it is provisionally assigned here as simply silicate or carbonate. The roughly 1,597 cm^−1^ peak closely matched the known graphitic (G) band observed on a sample from the Martian meteorite Sayh al Uhaymir (SaU008) calibration target in position and shape (Fig. 4c,d and Extended Data Fig. 5). In the calibration target, the Raman peak at roughly 1,599 cm^−1^ is known to be from macromolecular carbon^15,24^; thus, the 1,597 cm^−1^ peak is consistent with a carbon–carbon bond. This point on SaU008 similarly shows weak group 2 fluorescence at roughly 340 nm, although it seems to have lower intensity and longer emission wavelength than the point on Montpezat. Higher confidence in a specific Raman assignment would have been possible if the peak was detected at a greater signal-to-noise and seen at more than one point. Several nearby points showed possible peaks below the detection threshold.

The mean spectrum of points where the highest intensity group 2 features were detected on Garde (Séítah fm) yielded a Raman peak at roughly 1,403 cm^−1^ (Fig. 4e,f). These points were correlated to Raman detections consistent with olivine (823 cm^−1^), phosphate (960 cm^−1^) and carbonate (1,086 cm^−1^). Another possible peak in the mean spectrum was visible at roughly 1,540 cm^−1^, but was at the lower limit of detectable width (less than 3 pixels FWHM) so is unassigned. The roughly 1,403 cm^−1^ peak could be due to an organic compound, such as a C=O stretching vibration of an organic salt^25^. Organic salts are possible oxidation and radiolysis products of organic matter and have been indirectly detected on Mars previously^26^. Carbonyl groups and aromatic or olefinic carbon have been correlated with carbonate in a Martian meteorite^5^. Further work is continuing to rule out secondary modes of matrix minerals.

The group 2 fluorescence (roughly 335–350 nm) feature is consistent with a two-ring aromatic molecule, such as naphthalene. Alternatively, the emission spectra are also consistent with Ce^3+^ in phosphates, on the basis of laboratory data^27^. Both aromatic organics^6^ and Ce^3+^ have been associated with phosphate minerals in Martian meteorites^28,29^. With the data collected from Perseverance and our laboratory analyses, we cannot rule out a contribution from both inorganic and organic sources. The aromatic compounds would probably exist with some degree of chemical substitution or in specific steric configurations with respect to surrounding minerals, that would result in blue- or red-shifting from the expected fluorescence wavelengths for benzene and naphthalene. Red-shifting of fluorescence due to the formation of carboxylic acids on or near the aromatic ring is highly probable as these compounds are exposed to high energy radiation in an oxidative environment^30,31^, and previous studies of refractory organic carbon in Martian meteorites have shown carboxyl functionality^5,6^. It is highly probable that the detected fluorescence features, if organic, represent mixes of organic moieties rather than single emitters, and their overlapping spectra could cause variability in the apparent position and the FWHM of observed bands. This would align with the colocated detections of many fluorescence features on the same points. If the fluorescence is inorganic, the emissions could also be varied as Ce^3+^ luminescence is highly matrix dependent and affected by changes in mineralogy and mineral composition^19^.

### Group 3: single band at roughly 270–295 nm

Fluorescence bands between roughly 270 and 295 nm (FWHM of roughly 20 nm) were observed at many points in survey scans of three abraded (Guillaumes, Bellegarde, Alfalfa) and one natural target (Foux) in Máaz, and at few or no points in all other scans (Extended Data Figs. 1 and 2). On the targets with a substantial number of detections, points where group 3 fluorescence was detected often appeared clustered together on brown-toned, possibly iron-stained material (Extended Data Figs. 2 and 5). In many cases, this feature was colocated with the group 2 feature and was comparatively weaker in intensity (Fig. 2, and Extended Data Fig. 4). The average band centre position in natural targets (276.1 ± 0.8 nm) and abraded targets (276.1 ± 1.4 nm) in Máaz were similar. Given the few overall detections in Séítah, no quantitative comparison was possible. No clear mineral associations were detected with group 3 fluorescence in Máaz abraded targets, except Alfalfa. Here, fluorescence was associated with a broad Raman peak at roughly 1,040–1,080 cm^−1^, assigned to possible silicate^2,19^, and peaks at roughly 1,085–1,100 cm^−1^, assigned to carbonate^2,19^, at or near boundaries of black and grey grains. As with the group 2 feature, no clear textural associations were observed in natural targets, and no mineral signatures could be identified in the spectra. The group 3 (roughly 270–295 nm) feature is consistent with a single ring aromatic compound, such as benzene^16^; possible non-organic sources, such as silica defects, are discussed in the Methods.

### Group 4: roughly 290 and 330 nm features

**Extended Data Fig. 6:**
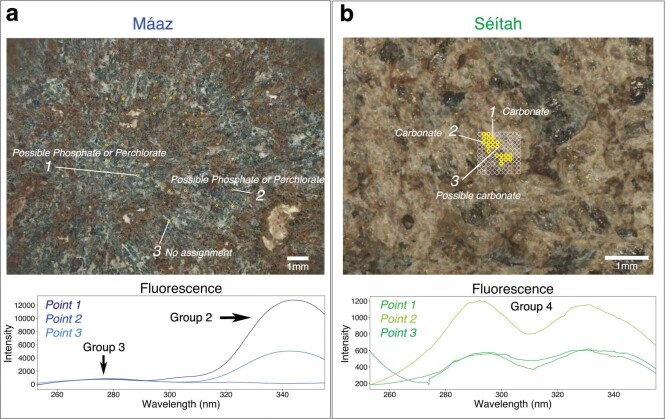
Group 3 Feature Mineral Association in Bellegarde and Group 4 Feature ~290 & 330 nm Feature and Mineral Associations in Garde. A) Colorized ACI of Bellegarde (Máaz fm.) HDR scan on sol 186 with laser overlay (gray = no fluorescence detected, yellow = fluorescence at ~270–295 nm detected). Three points of interest with Group 3 fluorescence are marked in white and correlate to the smoothed mean spectra below. Point 1 and 2 were co-located with a potential phosphate or perchlorate detection (~973, ~965 cm^−1^); Point 3 was not co-located with a mineral detection. In all of these points, high intensity Group 2 fluorescence was also detected. B) Colorized ACI of Garde (Séítah fm.) Detail centre 1 scan on sol 208 with laser overlay (gray = no fluorescence detected, yellow = fluorescence at ~290 & 330 nm doublet detected). Three points of interest with clear fluorescence are marked in white and correlate to the spectra below. All three points were co-located with Raman detections of potential carbonate (~1088, ~1080, ~1085 cm^−1^).

The feature with bands centred at roughly 290 and 330 nm was observed on two targets, Guillaumes (Máaz) and Garde (Séítah). In both, it was observed on several points in intergranular spaces; this was particularly apparent on Garde as previously reported^16^. On Guillaumes, group 4 fluorescence was not clearly associated with specific minerals. On Garde, it was colocated with Raman peaks at roughly 1,087–1,096 cm^−1^ and a broad peak at roughly 1,080 cm^−1^, assigned to carbonate and silicate, respectively^2,19^ (Extended Data Fig. 6). The relative intensities of the two peaks were not constant between points, indicating that they could be from several emitters. It is also possible that it is not a distinct category but simply a combination of group 2 and 3 species. The spectra are consistent with a one or two-ringed aromatic compound(s), though the possible inorganic sources of groups 2 and 3 may also apply to group 4.

## Relative abundance of organic compounds

The observed fluorescence response, if solely from organic molecules, can be used to provide a conservative estimate of concentration using a single ring aromatic (benzene) with a weak fluorescence cross-section and an assumed depth of penetration of 75 µm (refs. ^16,32^). This depth is a conservative estimate based on DUV transmission of more than 150 µm through Mars simulants^32^. Comparing the survey scans of the abraded surfaces, the localized concentrations are varied and range from 20 to 400 pg of organics where Alfalfa (Máaz) has some of the highest number of occurrences and localized concentrations. Furthermore, the bulk concentration in Máaz is an order of magnitude higher than in Séítah (roughly 20 versus 2 ppm).

## Diverse fluorescence across formations

**Fig. 5:**
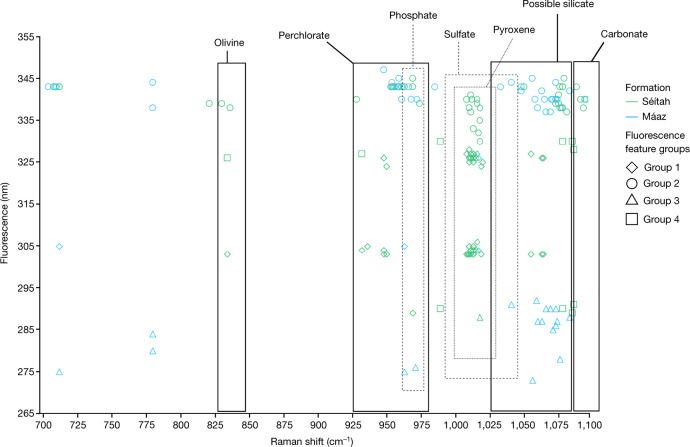
Summary of SHERLOC fluorescence-mineral associations across features and formations. Select mineral detections (Raman shift, cm^−1^) and their fluorescence features (*λ*_max_, nm) for abraded targets analysed using unsmoothed data from HDR and detail scans; both Raman and fluorescence data are measured on the same point. Máaz scans (blue) used between 250 and 500 ppp, yielding low signal-to-noise ratio (less than 2) in some cases that were not included; Séítah scans (green) all used 500 ppp, allowing for comparatively more Raman detections. Mineral classifications based on high confidence Raman detections of major peaks are indicated by boxed regions: olivine (roughly 825–847 cm^−1^)^2,19,26^, range of hydrated and dehydrated perchlorate (roughly 925–980 cm^−1^)^26,46^, phosphate (roughly 961–975 cm^−1^)^19,26,46^, pyroxene (roughly 1,000–1,026 cm^−1^)^19^, sulfate (roughly 990–1,041 cm^−1^)^2,19,26^, amorphous silicate (broad peak at roughly 1,020–1,080 cm^−1^)^2,26^ and carbonate (roughly 1,085–1,102 cm^−1^)^19,46^. Markers outside a boxed region do not have a mineral assignment. Disambiguation of overlapping regions can generally be resolved by consideration of minor Raman peaks (not marked here) and corroboration by other instrument(s) (for example, PIXL/SuperCam)^47^.

The Máaz and Séítah fm are two geologically and compositionally distinct formations that also show two different patterns of fluorescence. Following the hypothesis of the fluorescence being entirely organic in origin, these findings would indicate different bulk quantities of organic material, with Máaz having an order of magnitude more than Séítah. While colocations between organic features and minerals associated with aqueous processes were found in both formations, the colocation with primary igneous minerals was different. The group 2 feature was associated with olivine at many points in all Séítah targets and to pyroxene in two Máaz targets (Fig. 5). This suggests several mechanisms of synthesis or preservation, which may be at least partially unique to each formation. A similar pattern of organics associated with pyroxene and olivine has been shown in studies on meteorites ALH84001, Nakhla and Tissint. In these cases, the organic material has been shown to be synthesized in situ^5^. Further observation of the cored samples is needed to confirm the provenance and formation mechanism of this material.

Previous findings indicate that the two formations underwent different alteration processes. Máaz seems to be aqueously altered basaltic rock that contains Fe^3+^ bearing alteration minerals^33^. Séítah is proposed to be an olivine cumulate^13^ altered by fluids at low water to rock ratios^32^, and contains mafic minerals that have higher abundances of total FeO than Máaz rocks^34^. Owing to the presence in Máaz of Fe^3+^ bearing minerals, which can attenuate the fluorescence response^35^, we would expect fewer and lower intensity fluorescence detections than in Séítah. However, our observations demonstrate the opposite, with Máaz targets having more fluorescence detections and highest localized fluorescence intensities. If the fluorescence is organic, this demonstrates a correlation of organics occurrence and abundance with the degree of water-driven alteration and suggests that these signatures are driven by synthesis and/or transport mechanisms rather than meteoritic deposition, which would probably affect both formations in a similar manner. The concentrations of organics associated with more aqueously altered surfaces are consistent with known bulk concentrations of organics observed in Martian meteorites at roughly 11 ppm^1^ and in situ analysis performed by Curiosity rover in Gale crater that indicated organics concentrations from roughly 7 ppb to 11 ppm^2^.

The two formations also showed different types of fluorescence features. Whereas the group 1, 2 and 4 features were detected in both formations, Séítah showed a near-complete absence of group 3 features. This could indicate selective synthesis or preservation mechanisms that favour the organics associated with the longer wavelength fluorescence or a degradation process that only affected the group 3 associated organic molecules. The group 2 feature was most frequently detected in both formations, but showed differences in the abraded targets in Máaz and Séítah. Although the average band centre positions of the group 2 detections in both units were similar (Máaz 344.1 ± 1.5 nm; Séítah 343.1 ± 0.5 nm), the range of band centres in abraded Séítah targets was narrower (roughly 340–345 nm), whereas the band centres in abraded Máaz targets were more broadly distributed (roughly 338–349 nm).

## Potential mechanisms affecting organic matter

The four fluorescence features observed on the ten targets interrogated by SHERLOC each show varying degrees of mineral association and spatial patterning, suggesting that these features may originate from more than one mechanism of formation, deposition or preservation. Two of the features, groups 1 and 4, were highly localized to specific minerals, whereas the other two features were associated with several minerals and more broadly distributed. Continuing the organic hypothesis, the clearest association between a specific organic detection, mineral detection and texture was the group 1 feature found on Bellegarde and Quartier associated with white sulfate grains (Fig. 3). One possible mechanism consistent with this association is abiotic aqueous organic synthesis. Aromatic molecules, including sulfur-containing species, associated with sulfate have been found in Tissint, Nakhla and NWA 1950 (ref. ^5^) and were proposed in these cases to be the result of electrochemical reduction of aqueous CO_2_ to organic molecules due to interactions of spinel-group materials, sulfides and a brine. Organics in ALH84001 have been shown to be produced during carbonation and serpentinization reactions, indicating that several abiotic organic synthesis mechanisms can occur on Mars. Alternatively, this organic-mineral association could be the result of mineral-mediated selective preservation of transported organic compounds in sulfate. Previous work has shown that sulfates, including gypsum and magnesium sulfate, can protect organic molecules within their crystal lattices from UV radiation and oxidation^36^ and terrestrial evaporitic sulfate minerals can preserve organic material over geological timescales^37,38^.

The group 2 feature was the most frequently detected across all target types and formations, suggesting that a common synthesis, deposition, preservation or alteration process was responsible. Previous literature has implied that primary organic carbon is potentially ubiquitous in Martian basaltic rocks and there may be an abiotic reservoir of organic carbon on the planet^39^. Although most of the mineral associations of the group 2 feature were common across both formations, the association with pyroxene in Máaz targets and olivine in Séítah targets suggests that potentially distinct mineral-mediated processes influenced these organics (Fig. 5). These could include formation of an aromatic radiolysis product by means of a mineral-mediated alteration reaction^30,31,40,41^. The paucity of fluorescence detections and near absence of group 3 detections in Séítah suggest that there was different synthesis or deposition in this formation or the organic compounds were more thoroughly degraded. The relative ages of the two units are such that increased degradation in Séítah would have to be due to another phenomenon, such as accelerated erosion processes or potential brief exposure to a more acidic fluid than in Máaz that could affect organic matter.

In natural targets, fluorescence detections do not seem to correlate with morphological features or textures, which is consistent with the aromatic emitters present within the ubiquitous Martian dust. Meteoritic infall and interplanetary dust particles transport organic molecules to the surface of Mars, which are subsequently oxidized^42,43^. The presence of dust would also explain the fewer detectable signals on natural targets, as it may absorb or scatter incident light.

## Conclusions

Samples analysed in two formations within Jezero crater yielded detections by both fluorescence and Raman spectroscopy consistent with organic material that is colocated with specific mineral assemblages. The general spatial correlation between these detections and minerals that have undergone substantial aqueous processing suggests that organic molecules may have been abiotically aqueously deposited or synthesized within these altered volcanic materials within the crater floor. Differences in the nature and distribution of organic molecules in the formations would indicate that different aqueous alteration or deposition processes occurred, possibly contributing to the diversity of organic matter still present. The confirmation of organic origin and specific identification of these molecules will require samples to be returned to Earth for laboratory analysis. However, these results indicate a more complex organic geochemical cycle may have existed than has been described from previous in situ measurements on Mars, as evidenced by several distinct pools of possible organics. In summary, key building blocks for life may have been present over an extended period of time (from at least roughly 2.3–2.6 Ga, ref. ^44^), along with other as yet undetected chemical species that could be preserved within these two potentially habitable paleo-depositional settings in Jezero crater.

## Methods

### Further observations for group 2 fluorescence features

The overlap between the Raman signals from dehydrated perchlorates and phosphates coupled with low signal-to-noise made distinguishing between these assignments challenging in some cases. The key difference in mineral associations was that in three Máaz fm targets (Montpezat, Bellegarde and Alfalfa), the group 2 fluorescence feature was also associated with possible detections of pyroxene. By contrast, in the Séítah fm, this feature was associated with a possible detection of olivine in at least one point on each target (Extended Data Table 2).

Group 2 fluorescence bands in the natural targets (Máaz fm.) showed a similar shape to those in abraded targets, but the average band position in survey scans was 346.1 ± 2.0 nm, although it may be higher and obscured as it overlaps with the edge of the SHERLOC spectral range. This feature was not correlated to any specific texture. No identifiable Raman bands were detected on these targets, probably due to lower pulses per point (ppp) in the performed scans as well as signal attenuation due to out-of-focus regions and a dust layer; this precluded mineral identification. Signal attenuation aligns with the lower average intensity observed with the group 2 feature on natural targets (188 ± 42 counts) in comparison to dust-free abraded targets.

### Potential non-organic luminescence

Luminescence can be caused by non-organic sources as well as organic; however, excitation in the deep UV (less than 250 nm) has the advantage of being in the wavelength range to resonantly excite one- and two-ring aromatics and to avoid most of the interfering luminescence responses from rare earth ions. Nevertheless, the features of the presented dataset (including mineral associations, spatial distribution, frequency of detection, maximum lambda value (lambda max) of the emission bands, and context from previous Mars missions and Martian meteorites) should be compared in the context of each proposed source.

Fluorescence in inorganic minerals, such as feldspars^46^, can be due to emitters such as rare earth elements (REEs), or lanthanides and other metals within a mineral matrix that can act as activators^19^. REEs, in most cases, have emissions at wavelengths higher than the SHERLOC spectral range (that is, more than 360 nm)^19,48^. The most relevant REE to the reported detections is cerium, which can generate emissions within certain minerals in the detection range of SHERLOC. Under 266 nm excitation, Ce^3+^ in phosphates has been reported to emit roughly 340 nm luminescence^49^ that resembles some group 2 detections. In the dataset presented here, group 2 fluorescence is not always associated with a Raman identification of a phosphate mineral phase. However, Raman scattering of phosphates is not resonantly enhanced with the SHERLOC DUV laser, so the lack of a Raman detection colocated with 340 nm fluorescence does not preclude Ce^3+^ in phosphates as the source of the roughly 340 nm emission. The emission spectra of Ce^3+^ is highly matrix dependent and changes in mineralogy and mineral composition can notably affect the emission profile^19^. As shown in Fig. 5, the observed fluorescence is associated with a variety of minerals from aqueous processes that include sulfates, phosphates and carbonates, and is similar in position and shape regardless of association. Simple aromatic organic molecules can be preserved in these phases, and therefore are also potential sources for the reported fluorescence. However, it is possible that both organic and inorganic sources, or inorganic sources alone, contribute to the group 2 signals, as REE-bearing phosphates and organics preserved in phosphates have both been reported within Martian meteorites^50,51^.

In a laboratory study of synthetic ceric sulfate decomposition, strong photoluminescence emissions were reported^52^. In this study, both laboratory-synthesized and commercial ceric sulfate were heated to 500 °C for 16 h, then observed with a spectrofluorimeter. Ce^3+^ in both pentahydrated sulfates and anhydrous sulfate yielded double peaked emissions, at 319/339 and 322/339 nm, respectively. This latter observation aligns with other photoluminescence studies^53^. The closest emissions of cerium within sulfate reported in literature (at 304/327 nm), to the authors’ knowledge, is in a study of synthetic heat-treated anhydrite doped with Ce^3+^ and observed with cathodoluminescence^54^. There is an unexplained 12–13 nm difference in emissions of synthetic Ce-doped anhydrite and Ce^3+^ in natural anhydrite from many locations also measured in this study, indicating that the synthetic sample may not be the most accurate comparison to our dataset. As such, further laboratory analyses on both natural and synthetic cerium-containing sulfate samples are continuing. The lambda max of luminescence emission of cerium in sulfates is expected to shift on the basis of the hydration state of the mineral^52^. SHERLOC observed sulfates in different states of hydration, for instance on the several observations of the Quartier target, yet the observed fluorescence remained consistent in lambda max. Given the reported emissions of several organic molecules under DUV excitation (Extended Data Fig. 3) in this range, it seems likely that one or more of these molecules may be present in the sulfate minerals. The presence of organics could also possibly explain the Raman detections between roughly 1,300 and 1,650 cm^−1^. Finally, in the dataset presented here, group 1 was associated with sulfates in all cases; however, many points across targets showed clear Raman peaks of sulfates without the colocated fluorescence signal. This heterogeneity also aligns with the expected patterns of organics distribution. Further examination of the Quartier scans through more detailed analysis and laboratory comparisons is currently underway.

A subset of the signals in group 3 (roughly 281 nm) are also consistent with luminescence associated with defects in irradiated silica caused by oxygen deficiency centres^55^. However, we do not anticipate that the SHERLOC laser would create such defects, given the high power required to do so. Furthermore, we do not see a clear increase in detections at roughly 281 nm in higher ppp scans in comparison to low ppp, which would be expected if SHERLOC’s laser was inducing such damage. Investigation of other mechanisms (for example, radiation) that would cause localized silica defects that could produce luminescence consistent with group 3 features is continuing.

### Future possibilities for Mars sample return

The potential detection of organic molecules by SHERLOC in the abraded targets marked the corresponding cores as high priority for sampling during the crater floor campaign. If these samples are returned to terrestrial laboratories, a more diverse suite of tools can be used to study the samples, including at higher spatial resolution and with much greater specificity and sensitivity. The organic material and mineral relationships can be interpreted within the context of their original locations and stratigraphy, unveiling new insights into organic geochemical cycling on Mars.

### SHERLOC spectroscopy general operations

The use of a DUV wavelength may enable more sensitivity to aromatic organic molecules in complex matrices. At 248.6 nm wavelength excitation, a 10 to 1,000-fold increase in Raman scattering is provided by resonance and preresonance with aromatic organic molecules that have a large absorption cross-section. Measured Raman intensities are governed by both their Raman cross-sections and also the number of molecules excited. Transparent minerals with high scattering cross-sections can lead to large intensities whereas relatively few organic molecules in resonance with the SHERLOC laser can lead to similar intensities. Measured fluorescence intensities of mixtures are affected by their quantum yields but also self-absorption. Förster energy transfer can result in the measured intensity of only a single fluorophore even though a mixture of several species exists. Analysis of both fluorescence and Raman data can yield unique insight into mixtures of minerals and organics.

SHERLOC spectroscopy measurements are colocated with 1,648 × 1,200-pixel ACI autofocus full-frame images and placed on the desired target at a 48 mm standoff distance. Activities are constrained by the time of day the laser is operating, optimizing the temperature of the spectrometer CCD to be below −20 °C and reducing contributions from ambient light. Of the 14 instances SHERLOC spectroscopy has run on the surface of Mars to date, there was only one activity that occurred slightly outside this optimal temperature range (the first abraded target, Guillaumes run on sol 161). This temperature constraint to generate valuable science data for SHERLOC means that it is optimal for SHERLOC spectroscopy to be run in the evening, after 20:00 (or early in the morning, but evening is preferred). SHERLOC spectroscopy was conducted on natural samples at midday and abraded samples in the evening, after local sunset, with the abovementioned exception of Guillaumes on sol 161. The robotic arm is capable of placing SHERLOC within 12 mm of a targeted location; SHERLOC’s internal scanning mirror has a positioning error of less than 22 μm at the target. The spectrometer has an estimated uncertainty of ±5 cm^−1^ (±0.004 nm) in the 700–1,800 cm^−1^ region, on the basis of the calibration performed on sols 59 and 181. SHERLOC spectroscopy on natural and abraded targets has evolved since the initial natural surface measurement on sol 83. In general, there are two standard suite measurements, with slight modifications where necessary, for SHERLOC spectroscopy and ACI imaging scans: (1) HDR and survey scans, ACI four-image mosaic, ACI 31-image *z*-stack and (2) detailed scans, which are usually coupled with a survey scan run before the detail scans, for context. In this study, spatial correlations, histograms and average number of detections of fluorescence were conducted using survey scans; mineral-textural-organic correlations were performed using HDR and detailed scans. In the cases of two sols of observation on the same target, the following sols observations were used: Guillaumes 161, Quartier 293 and 304 and Dourbes 257 and 269. Two survey scans were performed on Guillaumes, Dourbes and Quartier. Sol 141 imaging on Foux had an incomplete overlap of WATSON imaging and SHERLOC spectroscopy mapping.

### SHERLOC spectroscopy sequences

#### Natural targets

HDR scans consisted of three sets of 100 spectra, coarse-spaced (780 µm step size), 7 × 7 mm^2^ scan area, at high ppp (100 ppp for the first two scans, 300 ppp for the final scan). The first natural sample, Nataani, uplinked on sol 83, had 5, 50 and 100 ppp. The survey scan consisted of one scan of 1,296 spectra, 144 µm step size, 5 × 5 mm^2^ scan area at low ppp; typically, 15 ppp, but 10 ppp and a step size of 200 µm was used for Nataani.

#### Abraded targets

The first abraded target, Guillaumes, followed the typical HDR scan sequencing, and consisted of three sets of 100 spectra; coarse-spaced (780 µm step size); 7 × 7 mm^2^ scan area; 100, 100 and 300 ppp followed by a survey scan of 1,296 spectra; 144 µm step size; 5 × 5 mm^2^ scan area and 15 ppp. In the targets analysed after Guillaumes, HDR scans were changed to two maps of 250 ppp (that is, 250/250) but conserved the total number of laser pulses (500), producing two 50 spectra maps for a total of 100 spectra when combined. The abraded samples also universally used a high laser current (25 A compared to the previous natural surface targets, which were shot at 20 A). When analysing the target, Garde, we had an option to use detailed mode scans for the first time. The initial scan on Garde on sol 207 used the standard suite HDR and survey scans. On sol 208, we did two sets of 50 spectra, 100 µm step size, 1 × 1 mm^2^ scan area and 500 ppp detailed scans. Although the survey scan was not included in sol 208, it became standard to include for subsequent detail scans (for example, Dourbes on sol 269 and Quartier on sol 304). The scan start position for all HDR and survey scan is at the centre, whereas for the detail scans the scanner starts in the corner or offset position.

### SHERLOC imaging operations

The two imaging systems, WATSON and ACI, are mounted atop a rotatable turret on the rover arm and are used during each SHERLOC observation. They are not coboresighted but the resulting images can be registered and overlaid to provide colour and textural information for a single target. WATSON acquires 1,600 × 1,200-pixel colour images of targets of interest from 2.5–40 cm standoff distances to provide broader context within the rock and outcrop. ACI images are always taken before spectroscopy and begin with two 256 × 256-pixel autofocus subframe and full-frame images. Further contextual imaging to support SHERLOC spectroscopy and correlation to images taken by other instruments, spectroscopy operations typically include a four-image ACI mosaic and a 31-image *z*-stack. The timing and lighting conditions of these products have been adjusted accordingly over the course of the ten targets (and 14 individual sample measurements) that SHERLOC has investigated. Typical operations for LED lighting are to take ACI images with all white LEDs turned on. Dourbes (sol 257) was the first time we had experimented with different lighting conditions on a target. For subsequent standard suite measurements, this update to the LED configuration (different group LEDs on and the use of UV LEDs) became a standard part of the sequences. The scanner is in the home position for the acquisition of the *z*-stack, which provides surface topographic relief when the in-focus images are assessed on the ground. The scanner is in the mosaic position for acquisition of the mosaic.

### Abrasion operations

Each selected target studied after sol 141 was abraded using the rover’s abrasion tool before SHERLOC observation. This tool grinds away the upper layer of rock, cuttings of which are then removed using the gaseous dust removal tool to reveal a fresh surface for analysis^14^. The resulting abrasion patch is a 45 mm diameter circle with a depth of 8–10 mm.

### Spectral data processing

Unsmoothed data without outlier removal were used to determine intensities and band positions. Preliminary spectral data processing was performed using an open-source software package named Loupe developed at the NASA Jet Propulsion Laboratory by K. Uckert. This software enables dark frame subtraction, laser normalization and selection of regions of interest (ROI), as well as the correlation of individual spectra to locations on the ACI image on the basis of the scanning mirror positioning. Exported Loupe data were then further processed using custom Python scripts, Microsoft Excel and Spectragryph^52^. These were used to perform baseline subtraction, outlier removal, peak detection and median smoothing in a semi-automated manner (the last only for fluorescence data in Fig. 4 and Extended Data Fig. 3). Outliers, generally caused by cosmic rays or charge buildup on the detector, were removed through subtraction and then the remaining data were interpolated across the spectrum. Requirements for fluorescence peak detection included FWHM of at least 100 pixels and more than five times the neighbouring background signal estimated by measurement in Loupe. A 10/1 signal-to-noise ratio was required for quantification, which may have excluded a small number points with actual signal but was deemed a robust criterion for accurate measurement of lambda max and FWHM. Fluorescence spectra used in Figs. 2–4, Extended Data Table 2 and Extended Data Figs. 4 and 5 were smoothed using the Savitzky–Golay algorithm with parameters manually tweaked after comparison to non-processed spectra. This was performed using the SciPy Python package^53^. This algorithm is known to introduce boundary artefacts^45^, which can be seen less than 270 nm in several spectra that are not representative of the true data. Fluorescence data were also fitted in Igor64 (Wavemetrics) to allow for measurement of lambda max and FWHM. Bands were fitted using Gaussian or exponentially modified Gaussian functions; baselines were fitted using constant, linear or cubic functions on the basis of visual analysis and chi-squared goodness-of-fit values. For cases in which the fluorescence band was cut off by the edge detector, such as in group 2, the band was always assumed to be symmetric beyond the cut off. For Fig. 5, lambda max and FWHM of each fluorescence spectrum was measured before association to a Raman signal (and possible mineral association) was considered, to maintain objectivity and avoid bias. Requirements for Raman peak detection included FWHM of at least 4 pixels and more than twice the neighbouring (10 pixels) background signal intensity estimated by measurement in Loupe. This width threshold was selected on the basis of the spectral resolution of the instrument (roughly 3–4 pixels)^54^. FWHM of Raman spectra in Fig. 4 were baselined using the airPLS algorithm^55^ implemented in Python. Unsmoothed Raman data were fitted using the Multipeak Fit package in Igor64 (Wavemetrics), which enabled peak identification and fitting as well as baseline fitting and chi-squared value approximation. The signal-to-noise ratio for SHERLOC data from the rock surfaces was lower as expected than on calibration targets (Fig. 4); in applicable cases, data from several points were averaged to remove the impact of cosmic rays and improve signal.

### Image processing

Image processing on both WATSON and ACI products was performed using a Python script to register several images for a single target to create an overlay. The script uses the OpenCV library built in classes to implement BRISK keypoint detection and a FLANN-based matcher to match keypoints to generate the overlays. ACI ECM products and WATSON ECM or ECZ (roughly 4 to 10 cm standoff) images were used in all cases. Colourized ACI products used for correlating spectral, colour and textural information were generated as previously described^16^. Small artefacts of bright colours are visible in these colourized images in certain cases.

### SHERLOC analogue instrument data

Reference spectra presented were collected on two laboratory instruments, Brassboard (Jet Propulsion Laboratory) and MORIARTI (Mineralogy and Organics Raman Instrumentation for the Analysis of Terrestrial Illumination) (University of Pittsburgh), that are analogues of SHERLOC modified to operate under terrestrial ambient conditions. Brassboard configuration and operations are described in previous literature^20^. MORIARTI is a custom DUV Raman microscope coupled with several spectrometers to cover the entire Raman and fluorescence (UV and visible light) spectral range. Samples can be illuminated with either a Coherent Industries Innova 300 FreD frequency-doubled Ar+ laser (248.3 nm, roughly 10 mW average power) or a Photon System NeCu laser (248.6 nm, roughly 20 μJ per pulse, 80 Hz). Laser light passes through a 248.6 nm laser clean up filter before being focused onto a turning prism and directed onto the sample as a roughly 120 μm diameter spot. Scattered and emitted light is collected in a 180° backscatter geometry using an f1.25 reflective cassegrain objective and passes through a Semrock 248 nm long-pass filter before entering one of the spectrometers. For Raman, light is dispersed from 250 to 278 nm to a resolution of 9 cm^−1^ inside an f/6.8 Czerny–Turner spectrograph and focused onto a Princeton Instruments liquid nitrogen cooled Pylon 400B CCD. For UV fluorescence, light is dispersed from 180 to 350 nm to a resolution of 0.5 nm by a custom Ocean Optics QE Pro spectrograph. For visible fluorescence, light is dispersed from 250 to 1,100 nm, to a resolution of 1.5 nm by an Ocean Optics HR4000 spectrograph. The sample can also be illuminated by a halogen white light, in which it is imaged onto a 1.6MP Thorlabs CMOS camera.

## Online content

Any methods, additional references, Nature Portfolio reporting summaries, source data, extended data, supplementary information, acknowledgements, peer review information; details of author contributions and competing interests; and statements of data and code availability are available at 10.1038/s41586-023-06143-z.

## Figures and Tables

**Extended Data Table 1:**
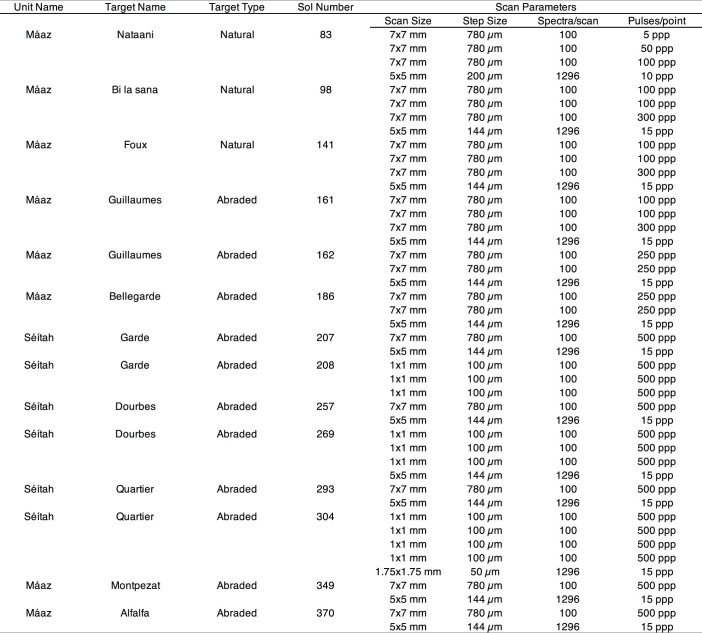
Overview of Scan Parameters Across Targets Performed by SHERLOC in the Crater Floor Campaign

Each scan analyzed in this study is listed here with its respective parameters; including size in millimeters, step size in microns, number of spectra per map, and pulses per point (ppp).

**Extended Data Table 2:**
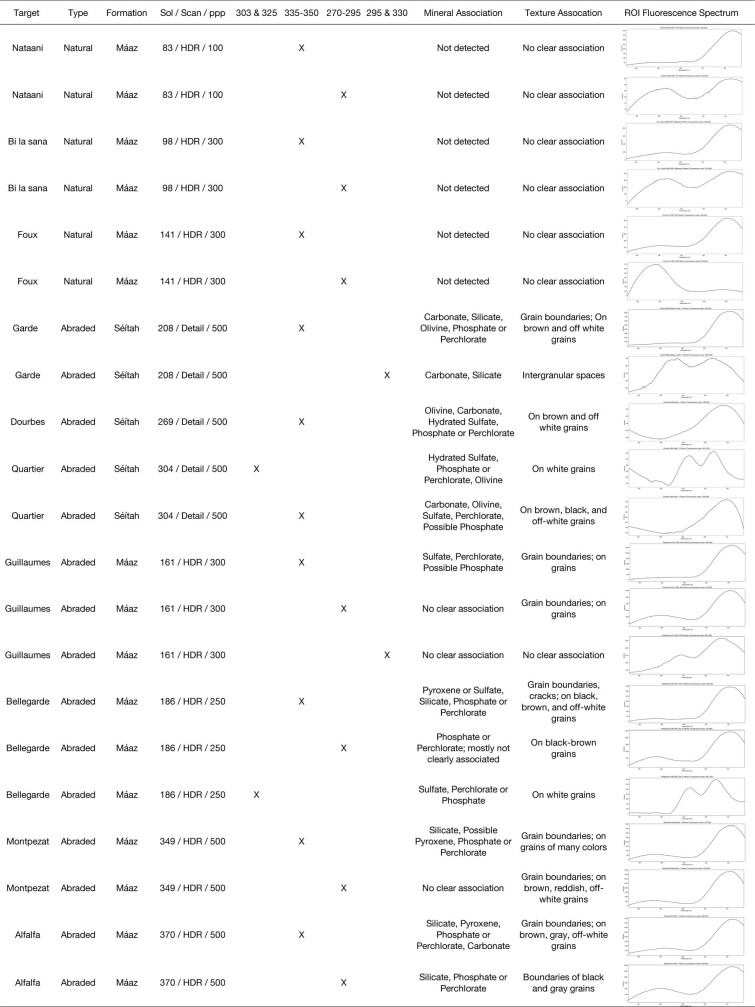
Summary of Fluorescence Features

A summary table indicating fluorescence, mineral, and textural observations for each target alongside representative fluorescence spectra for region of interest (ROI) for the detected feature group, defined by all the points within the scan that exhibited spectra with that group. All data shown here is compiled from the highest signal-to-noise ratio scan performed on each target.

**Extended Data Table 3:**
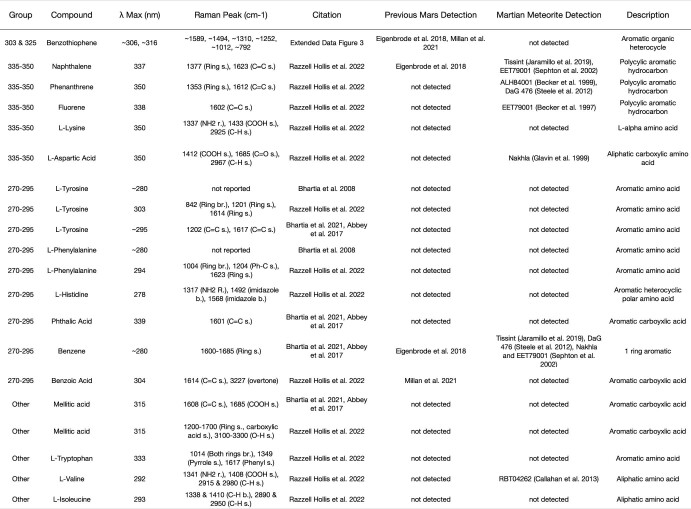
Relevant Deep UV (248.6 nm) Raman and Fluorescence Bands and Assignments for Organic Molecule Detection

The four fluorescence feature categories were developed based on known patterns of fluorescence of one-ring and polycyclic aromatic compounds. Each compound of relevance is presented with known fluorescence bands and Raman peaks, citation^15–17,46,56–61^, description, and previous detection on Mars^3,4^ or within Martian meteorites^21,47–51^.

## Data Availability

The data used for this study are released on the Planetary Data System (PDS) at https://pds.nasa.gov/. Data from the SHERLOC instrument are accessible at https://pds-geosciences.wustl.edu/missions/mars2020/sherloc.htm. Spectral data are organized by sol number and accessible in csv format at https://pds-geosciences.wustl.edu/m2020/urn-nasa-pds-mars2020_sherloc/data_processed/. Fundamental data record image data acquired by the ACI are organized by sol number and accessible in IMG format at https://pds-imaging.jpl.nasa.gov/data/mars2020/mars2020_imgops/data_aci_imgops/sol/. Fundamental data record image data acquired by the WATSON are organized by sol number and accessible in IMG format at https://pds-imaging.jpl.nasa.gov/data/mars2020/mars2020_imgops/data_watson_imgops/sol/.

## Code availability

All code used for image and data processing in this manuscript uses open-source libraries or previously published methods described herein. The code for ACI colorization is available under the Apache 2.0 licence at https://github.com/nasa-jpl/ACI-colorization. Loupe software is open source under the Apache 2.0 licence and available at https://github.com/nasa/Loupe.
